# Modeling of Fenton Reaction for the Oxidation of Phenol in Water

**DOI:** 10.1155/JAMMC.2005.31

**Published:** 2005

**Authors:** Olivier Chedeville, Ayse Tosun-Bayraktar, Catherine Porte

**Affiliations:** 1 Laboratoire de Chimie Industrielle Génie des Procédés pour l'Environnement et la Chinnie Fine du CNAM (EA 21) 2 rue Conté Paris cedex 03 75003 France

## Abstract

Phenol is an organic pollutant found in various types of industrial wastewater. Due to its bactericidal properties, it is difficult to eliminate it by classic treatment methods. In this work, the degradation of this compound by Fenton reaction at mild temperature and pressure conditions is studied. An experimental design was applied in order to quantify the influence of operating parameters on the efficiency of this method. The field of study was defined between 20 and 50^^^C for the temperature, 1 and 4 g L^-1^ for the phenol concentration, 10 and 28 for the H_2_O_2_ to phenol molar ratio, and 0.02 to 0.08 for the Fe(II) to phenol concentration ratio. It was shown that the temperature and the amount of catalyst have a strong influence. A model giving the decrease of COD was established. The COD decrease was between 40% and 72% and phenol had totally disappeared.


Journal of Automated Methods & Management in Chemistry, **2005** (2005), no. 2, 31–36
© 2005 Hindawi Publishing Corporation

### Modeling of Fenton Reaction for the Oxidation of Phenol in Water

Olivier Chedeville, Ayse Tosun-Bayraktar, and Catherine Porte

*Laboratoire de Chimie Industrielle, Génie des Procédés pour l'Environnement et la Chinnie Fine du CNAM (EA 21), 2 rue Conté, 75003 Paris cedex 03, France*

*Received 21 June 2004; Accepted 26 October 2004*

Phenol is an organic pollutant found in various types of industrial wastewater. Due to its bactericidal properties, it is difficult to eliminate it by classic treatment methods. In this work, the degradation of this compound by Fenton reaction at mild temperature and pressure conditions is studied. An experimental design was applied in order to quantify the influence of operating parameters on the efficiency of this method. The field of study was defined between 20 and 50°C for the temperature, 1 and 4 g $L^{-1}$ for the phenol concentration, 10 and 28 for the $H_2O_2$ to phenol molar ratio, and 0.02 to 0.08 for the Fe(II) to phenol concentration ratio. It was shown that the temperature and the amount of catalyst have a strong influence. A model giving the decrease of COD was established. The COD decrease was between 40% and 72% and phenol had totally disappeared.

### 1. INTRODUCTION

During its use in industrial processes, water may be polluted by different compounds. The effluents must be treated before dumping to limit the environmental impact. However, some compounds are difficult to eliminate because of their high chemical stability and/or their high concentration.

Phenol, which is largely used in chemical industry, is one of these compounds. In France, phenol in liquid effluents amounted to 54 tons in 2000 [1]. In addition to its organoleptic properties (it gives a characteristic taste and odor to water even at low concentrations), it is also a bactericide. So its treatment by classic water treatment methods (including a biological treatment step) is difficult at high concentrations. Thus, its degradation by a specific method should be performed directly after its production before it is sent to the sewage treatment plants.

An efficient method may be the use of an advanced oxidation process: Fenton, photo-Fenton, $O_3$/UV, $O_3/H_2O_2$, $O_3$/UV/$H_2O_2$. Generally, these methods lead to high degradation ratios [2, 3, 4, 5].

This study is based on the use of the Fenton reaction. The objective of this method is the formation of hydroxyl radicals, $HO^{\cdot}$, through the decomposition of hydrogen peroxide $H_2O_2$ catalyzed by iron in the form Fe(II). These radicals, having a short lifetime, are some of the most powerful oxidizers in water and allow the degradation of a lot of organic compounds.

Previous studies showed that the concentrations of phenol, iron, and hydrogen peroxide, and temperature, and pH have a significant influence on the efficiency of this method. The aim of this study is to quantify the influence of these parameters by modeling the efficiency of the reaction measured by chemical oxygen demand (COD) decrease, expressed in percentage. The experiments were performed to a factorial experimental design.

### 2. PRELIMINARY CONSIDERATIONS

### 2.1. Fenton's reaction

$H_2O_2$ is largely used in oxidation processes. Its oxidizing properties are not only due to the presence of an active oxygen atom in the molecule, but also to its ability to participate in radical reactions, with the homolytic cut of the O−O bound leading to the formation of the $HO^{\cdot}$ radical. This ability is enhanced by the presence of compounds able to activate these radical decomposition mechanisms.

Fenton's reaction is based on the following principle: in the presence of Fe(II) as a catalyst, a succession of reactions induces the formation of hydroxyl radicals, used to oxidize the organic pollutant [6, 7]:

$$Fe^{2+} + H_2O_2 \longrightarrow Fe^{3+} + HO^{\cdot} + HO^{-} \quad (K = 76.5\ \mathrm{L\ mol^{-1} s^{-1}}). \tag{1}$$

Correspondence and reprint requests to Catherine Porte, Laboratoire de Chimie Industrielle, Génie des Procédés pour l'Environnement et la Chinnie Fine du CNAM (EA 21), 2 rue Conté, 75003 Paris cedex 03, France; Tel: +33 1 40272393; Fax: +33 1 440272069; E-mail: cporte@cnam.fr.

Table 1: Matrix of experiments ($A$) and experimental results ($Y$).

| $N$ | Reduced coordinates | | | | Real variables | | | | Response |
|---|---|---|---|---|---|---|---|---|---|
| | $X_1$ | $X_2$ | $X_3$ | $X_4$ | [Phenol] (g $L^{-1}$) | $n_{H_2O_2}/n_{phenol}$ | $[Fe^{2+}]$/[phenol] | $T$(°C) | $y$ = COD decrease (%) |
| 1 | −1 | −1 | −1 | −1 | 1 | 10 | 0.02 | 20 | 41.5 |
| 2 | 1 | −1 | −1 | −1 | 4 | 10 | 0.02 | 20 | 44 |
| 3 | −1 | 1 | −1 | −1 | 1 | 28 | 0.02 | 20 | 50 |
| 4 | 1 | 1 | −1 | −1 | 4 | 28 | 0.02 | 20 | 40 |
| 5 | −1 | −1 | 1 | −1 | 1 | 10 | 0.08 | 20 | 59 |
| 6 | 1 | −1 | 1 | −1 | 4 | 10 | 0.08 | 20 | 61 |
| 7 | −1 | 1 | 1 | −1 | 1 | 28 | 0.08 | 20 | 50 |
| 8 | 1 | 1 | 1 | −1 | 4 | 28 | 0.08 | 20 | 65 |
| 9 | −1 | −1 | −1 | 1 | 1 | 10 | 0.02 | 50 | 51.5 |
| 10 | 1 | −1 | −1 | 1 | 4 | 10 | 0.02 | 50 | 58 |
| 11 | −1 | 1 | −1 | 1 | 1 | 28 | 0.02 | 50 | 55 |
| 12 | 1 | 1 | −1 | 1 | 4 | 28 | 0.02 | 50 | 52.5 |
| 13 | −1 | −1 | 1 | 1 | 1 | 10 | 0.08 | 50 | 72 |
| 14 | 1 | −1 | 1 | 1 | 4 | 10 | 0.08 | 50 | 72 |
| 15 | −1 | 1 | 1 | 1 | 1 | 28 | 0.08 | 50 | 71 |
| 16 | 1 | 1 | 1 | 1 | 4 | 28 | 0.08 | 50 | 68 |

Fe(II) can be regenerated in the following way:

$$Fe^{3+} + H_2O_2 + H_2O \rightleftarrows FeOOH^{2+} + H_3O^+ \quad (K_{eq} = 3.1 \times 10^{-3}), \tag{2}$$

$$FeOOH^{2+} \longrightarrow HO_2^{\cdot} + Fe^{2+} \quad (K = 2.7 \times 10^{-3} s^{-1}), \tag{3}$$

$$Fe^{3+} + HO^{2\cdot} \longrightarrow Fe^{2+} + O_2 + H^+ \quad (K < 2 \times 10^3 \text{ L mol}^{-1} s^{-1}). \tag{4}$$

Firstly, hydroxyl radicals attack the phenol to form catechol and hydroquinone [8]. The decomposition of these byproducts then gives ring opened products such as glyoxalic, formic, oxalic, fumaric, maleic acids, which can be easily removed by biological treatment.

In order to avoid the decomposition of $H_2O_2$, reaction (2) must be inhibited by limiting the amount of $H_2O_2$ in the reaction medium. This can be achieved if it is introduced continuously. The pH of the reaction mixture, on the other hand, must be kept between 2 and 4, firstly in order to avoid the formation of Fe complexes that can decrease the efficiency of the catalyst and secondly to favor the formation of a stable and electrophilic structure via solvation of a proton by an $H_2O_2$ molecule [6].

### 2.2. Experimental design

The objective of an experimental design is to quantify the impact of the experimental factors on the efficiency of the treatment by establishing a mathematical model: efficiency = $f$(factor 1, factor 2, ...). Here, a first-degree model design was applied, and the relation obtained was (for $n$ variables)

$$y = a_0 + \sum_{i=1}^{i=n} a_i X_i + \sum_{i=1}^{n-1} \sum_{j>i}^{n} a_{ij} X_i X_j + \sum_{i=1}^{n-2} \sum_{j>i}^{n-1} \sum_{k>j}^{n} a_{ijk} X_i X_j X_k + \cdots, \tag{5}$$

where $y$ is the response of the system (decrease of COD), $X_i$ is the reduced coordinate of variable $i$, $a_i$ is the coefficient of the model.

The maximal value of the field of study was associated to the reduced coordinate +1, and the minimal value to the reduced coordinate −1.

For a 4-variable experimental design, 16 coefficients have to be determined. The experimental design consists in setting up a system of equations to find these coefficients by investigating the field of study of every variable. Hence, the resolution requires 16 different equations:

$$\begin{aligned} y_1 &= a_0 + a_1 X_{1,1} + \cdots + a_{1234} X_{1,1} X_{2,1} X_{3,1} X_{4,1}, \\ &\vdots \\ y_{16} &= a_0 + a_1 X_{1,16} + \cdots + a_{1234} X_{1,16} X_{2,16} X_{3,16} X_{4,16}, \end{aligned} \tag{6}$$

where $y_j$ is the response of the system to experiment $j$, $X_{ij}$ is the value of variable $i$ for experiment $j$.

The experimental design used for this study is presented in Table 1. The previous system of equations can be represented in a matrix shape as follows:

$$(Y) = (A) \cdot (a), \tag{7}$$

where $(Y)$ is the matrix of the results, $(A)$ is the matrix of the experimental design, and $(a)$ is the matrix of the coefficients.

As $({}^tM \cdot M)^{-1} \cdot ({}^tM \cdot M) = (I)$, by multiplying every member of the previous equation by $({}^tA \cdot A)^{-1} \cdot ({}^tA)$, the following equation is obtained:

$$(a) = ({}^tA \cdot A)^{-1} \cdot ({}^tA) \cdot (Y). \tag{8}$$

This relation allows all of the coefficients of the model to be determined.

### 3. EXPERIMENTAL SETUP

### 3.1. Procedure

The experiments were carried out in a thermostated batch reactor of 1 L equipped with a reflux condenser and a mechanical stirrer (Figure 1). A volume of 500 mL of solution of phenol and Fe(II) was introduced into the reactor under stirring at a constant temperature value. The hydrogen peroxide was introduced continuously by means of a peristaltic pump. The liquid samples, taken via the drain valve, were analyzed in order to follow the evolution of phenol concentration and COD degradation. Immediately before measurement, the excess of $H_2O_2$ remaining in the samples was removed by adding an equivalent amount of sodium sulfite.

### 3.2. Materials and methods of analyses

The catalyst in the form of $FeSO_4$, $7H_2O$, the phenol in the form of crystals, the 30% hydrogen peroxide, and the sodium sulfite came from VWR, Paris, France. Other compounds used for the determination of by-products were obtained from Sigma Aldrich, Lyon, France. All the solutions were prepared with distilled water.

The phenol and the by-products were analyzed by HPLC (model SS Wakosil II 5C18RS), equipped with a UV detector ($\lambda = 210$ nm) by using as a mobile phase a mixture of acetonitrile : water / 50 : 50 in a flow of 1 mL $min^{-1}$. The COD was measured by method 8000 of HACH with the spectrophotometer DR/2000.

### 4. EXPERIMENTAL RESULTS

### 4.1. Preliminary experiments

First a series of experiments was conducted to determine the limit duration of the reaction: it seems that after two hours, while the reaction was as slow as possible (low temperature and low concentration of catalyst) the COD value (Figure 2) does not change. A limit of two-hour duration was thus retained for all the experiments.

Furthermore, HPLC analyses showed that the phenol disappeared in the first minutes of the reaction, and that the main by-products formed were catechol, hydroquinone, oxalic acid, acetic acid, and formic acid. Small amounts of maleic acid and acrylic acid were also detected.

### 4.2. Experimental design

Four variables were retained for this experimental design: the initial phenol concentration ($X_1$), the ratio of the amount of hydrogen peroxide to that of phenol introduced ($X_2$), the ratio of the concentration of Fe(II) to the concentration of phenol ($X_3$), and the temperature ($X_4$). The injection duration for the peroxide was fixed after about 40 minutes and the pH was fixed at 3 at the beginning of the reaction by the addition of 1 M sulfuric acid. The fields of study were chosen according to the conditions usually met in the industry (Table 2). The response, $y$, of the system was the percentage of decrease of the COD, $y = 100 \cdot [(COD_{initial}/COD_{final})/COD_{initial}]$.

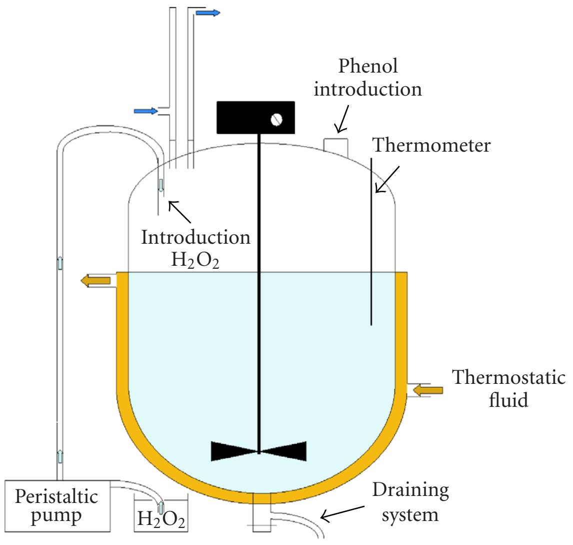

FIGURE 1: Reactor.

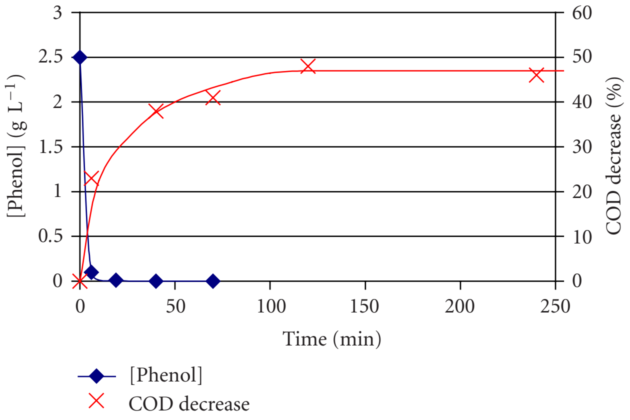

FIGURE 2: Evolution of phenol concentration and COD.

TABLE 2: Field of study of experimental parameters.

| Variable | Level −1 | Level +1 |
|---|---|---|
| $[Phenol]_0$ | 1 g $L^{-1}$ | 4 g $L^{-1}$ |
| $n_{H_2O_2}/n_{phenol}$ | 10 | 28 |
| $[Fe(II)]/[phenol]_0$ | 0.02 | 0.08 |
| Temperature | 20°C | 50°C |

The dilution effect due to the addition of $H_2O_2$ and a solution of sodium sulfite was taken into account. The response of each experiment is presented in Table 1.

The matrix resolution allowed to calculate the various coefficients of the model is shown in Table 3.

### 4.3. Repeatability and validation of the model

The experiment at the central point of the model (the value of the coordinates of all the variables is 0: $[phenol]_0 = 2.5$ g $L^{-1}$, $n_{H_2O_2}/n_{phenol} = 19$, $[Fe(II)]/[phenol] = 0.05$ and $T = 35°C$) has been repeated five times. The decreases of

Table 3: Coefficients of the model.

| | Direct terms | | Second-order interaction | | Third-order interaction | | Fourth-order interaction | |
|---|---|---|---|---|---|---|---|---|
| Constant term | $a_0$ | 56.9 | $a_{12}$ | −0.7 | $a_{123}$ | 2.0 | $a_{1234}$ | −1.2 |
| $[\text{Phenol}]_0$ | $a_1$ | 0.7 | $a_{13}$ | 1.1 | $a_{124}$ | −0.8 | — | — |
| $n_{H_2O_2}/n_{phenol}$ | $a_2$ | −0.5 | $a_{14}$ | −0.5 | $a_{134}$ | −2.0 | — | — |
| $[\text{Fe(II)}]/[\text{phenol}]_0$ | $a_3$ | 7.8 | $a_{23}$ | −0.8 | $a_{234}$ | 0.4 | — | — |
| Temperature | $a_4$ | 5.6 | $a_{24}$ | −0.4 | — | — | — | — |
| | | | $a_{34}$ | 0.4 | — | — | — | — |

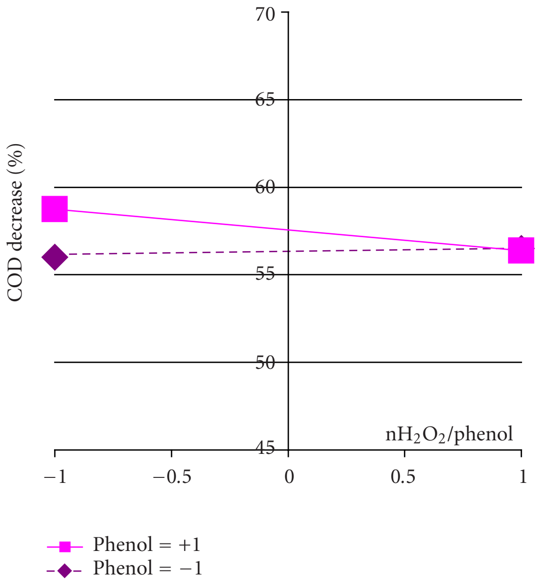

Figure 3: Interaction $X_1X_2$.

COD were respectively 57.8%, 58.4%, 56.8%, 57.7%, 58.5%. So, the average result of these experiments was 57.8% and the standard deviation was $\sigma_{exp} = 0.7$, which was in good agreement with the 56.9% predicted by the model and allowed to verify its linearity. The variance $\sigma^2$ on every coefficient was calculated according to the relation

$$\sigma^2 = \frac{\sigma_{exp}^2}{2^k} = 0.028, \tag{9}$$

where

$$\sigma_{exp}^2 = 0.46, \qquad k = 16 \text{ (number of experiments)}. \tag{10}$$

The standard deviation $\sigma$ on every coefficient is then 0.17. Given the value of the coefficients (Table 3), these were all significant.

### 5. DISCUSSION

### 5.1. Treatment efficiency

The average decrease of COD for all the experiments was of 56.9% ($a_0$) and some experiments showed decreases up to 70% (experiments 13, 14, 15). Furthermore, HPLC analyses showed that phenol disappeared during the first few minutes of the treatment. Oxidation by Fenton's reaction is an efficient method to eliminate phenol.

### 5.2. Influence of the variables

Table 3 shows that two of the first-order coefficients were relatively high ($a_3 = 7.8$ and $a_4 = 5.6$), whereas the other two were much lower ($a_1 = 0.7$ and $a_2 = 0.5$). According to these results, in the chosen field of study, the amount of catalyst and the temperature had an important influence on the efficiency of the method. The initial concentration of phenol and the quantity of hydrogen peroxide introduced were shown to have less impact on the final result.

Moreover, these coefficients indicate that the higher the temperature and the quantity of iron were, the more important the COD decrease becomes. The average decrease of COD was of 49% with low iron concentrations and of 64.7% with high concentrations. This could be due to the inefficient regeneration of the catalyst. Moreover, the average decrease was of 51.3% at 20°C and of 62.5% at 35°C.

It was different for the other two variables. The average decrease of COD was of 56.2% for $[\text{phenol}]_0 = 1\,\text{g L}^{-1}$ and of 57.5% for $[\text{phenol}]_0 = 4\,\text{g L}^{-1}$. Also, 57.3% of the COD were eliminated at low concentrations of $H_2O_2$ and 56.4% for high concentrations. These results show that it was not necessary to introduce hydrogen peroxide in excess since the reaction was not as effective in these conditions. In view of these results, the optimal value of the latter variable may even be lower than the low limit chosen.

### 5.3. Interactions between variables

The study of the interactions between variables shows the impact of one variable on another affecting the efficiency of the treatment. According to the values of the second-order coefficients, three interactions seemed to be significant: the interactions between $X_1$ and $X_2$ ($a_{12} = -0.7$), between $X_1$ and $X_3$ ($a_{13} = 1.1$), and between $X_2$ and $X_3$ ($a_{23} = -0.8$).

In Figure 3, the interaction between variables $[\text{phenol}]_0$ and $n_{H_2O_2}/n_{phenol}$ is presented. This figure shows that for low pollutant concentrations, the quantity of $H_2O_2$ did not have much effect on the efficiency of the treatment, but for high phenol concentrations, better results were obtained with a lower concentration of peroxide. This lack of efficiency can be attributed to a parasitic decomposition of $H_2O_2$ when it was in excess amount in the reaction medium.

There is also a strong interaction between variables $X_1$ and $X_3$. The previous results showed that the higher the

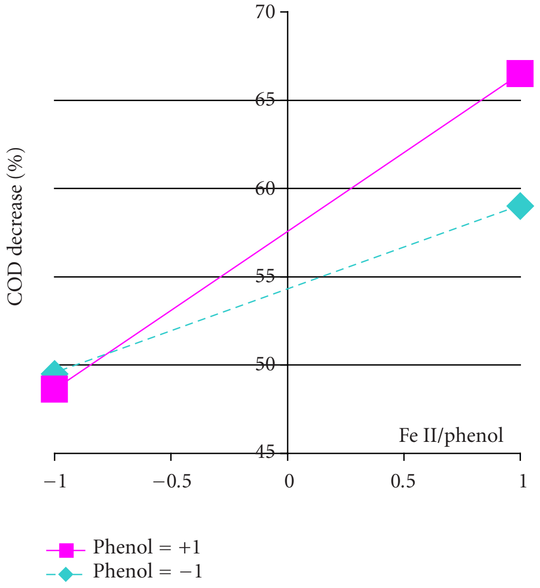

FIGURE 4: Interaction $X_1X_3$.

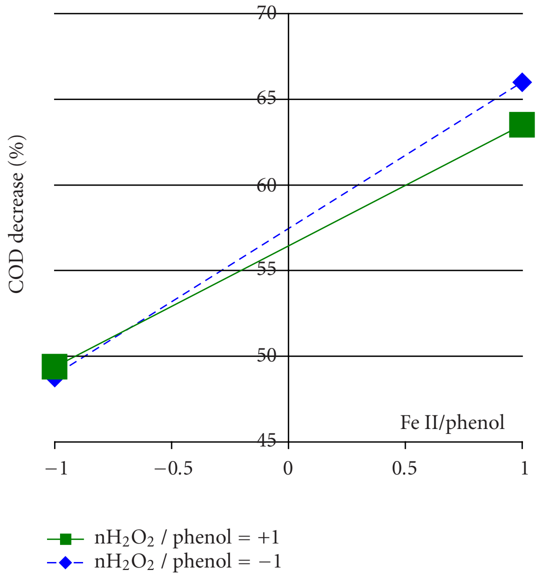

FIGURE 5: Interaction $X_2X_3$.

amount of catalyst was, the more effective the reaction was. Figure 4 shows that this effect was more significant when the concentration of phenol was high.

Figure 5 shows the interaction between variables $X_2$ and $X_3$. The effect was less significant here than for the previous interactions. Previous results had shown that the reaction was slightly more effective when there was less hydrogen peroxide present. The study of the interactions showed that the higher the concentration of catalyst was, the more significant this effect becomes.

Other interactions of the second degree were not significant.

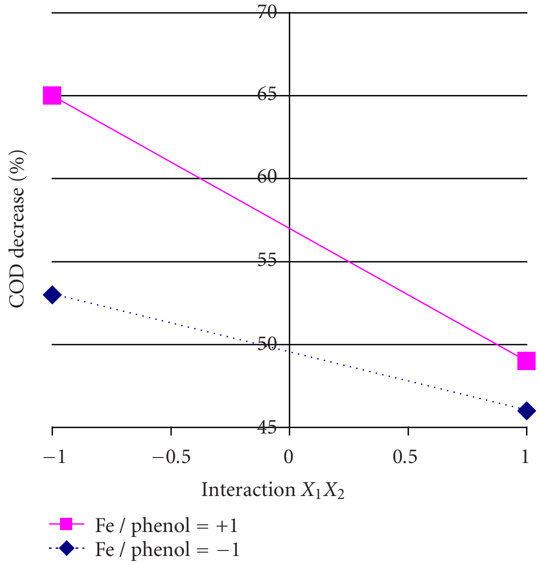

FIGURE 6: Interaction $X_1X_2X_3$.

Results indicate that two interactions of the third degree are not negligible: $a_{123} = 2$ and $a_{134} = -2$. Figure 6 shows the interaction between variables $X_1$, $X_2$, and $X_3$. It appears that the influence of the interaction $X_1X_2$ was enhanced by variable $X_3$.

### 6. CONCLUSION

The Fenton reaction is an effective method for the treatment of phenol: in the experimental conditions used, this pollutant was totally removed during the first few minutes of treatment, and, after two hours of treatment, a decrease of COD from 40% to 72% was obtained. Moreover, carboxylic acids of low molecular weight were detected. These can be easily removed by biological treatment.

An experimental design was applied to quantify the influence of four variables (temperature, concentrations of hydrogen peroxide, iron, and phenol) on the efficiency of the treatment. In the field of study considered, the dominating parameters for the COD decrease were temperature and iron concentration. The concentration of $[\text{phenol}]_0$ and amount of $H_2O_2$ were less influent. Moreover interactions between these variables (phenol and iron, phenol and hydrogen peroxide, and iron and hydrogen peroxide) were significant.

The model shows that for any given concentration of phenol, the value of the parameters which lead to the desired COD decreases. Other considerations (e.g., the cost of the treatment) can be used in order to find the optimal combination given the operating circumstances.

The model indicates that an excess of hydrogen peroxide does not improve the efficiency of the treatment. Moreover, analysis shows that mineralization is incomplete. So, there may exist an optimal value for the amount of $H_2O_2$, still lower than the minimal value chosen for the experimental design, which would increase the efficiency of the process. Further research to determine the optimal value could be carried out by using the method Uniplex.

### REFERENCES

[1] Ministère de l'Aménagement du Territoire et de l'Environnement, *Principaux rejets industriels en France—bilan de l'année 2000*, March 2002.

[2] W. Spacek, R. Bauer, and G. Heisler, "Heterogeneous and homogeneous wastewater treatment—comparison between photodegradation with $TiO_2$ and the photo-Fenton reaction," *Chemosphere*, vol. 30, no. 3, pp. 477–484, 1995.

[3] I. Akmehmet Balcioglu and M. Ötker, "Treatment of pharmaceutical wastewater containing antibiotics by $O_3$ and $O_3/H_2O_2$ processes," *Chemosphere*, vol. 50, no. 1, pp. 85–95, 2003.

[4] N. Kang, D. Soo Lee, and J. Yoon, "Kinetic modeling of Fenton oxidation of phenol and monochlorophenols," *Chemosphere*, vol. 47, no. 9, pp. 915–924, 2002.

[5] F. Luck, "Wet air oxidation: past, present and future," *Catalysis Today*, vol. 53, no. 1, pp. 81–91, 1999.

[6] M. Perez, F. Torrades, J. A. Garcia-Hortal, X. Domenech, and J. Peral, "Removal of organic contaminants in paper pulp treatment effluents under Fenton and photo-Fenton conditions," *Applied Catalysis B: Environmental*, vol. 36, no. 1, pp. 63–74, 2002.

[7] J. J. Pignatello and G. Chapa, "Degradation of PCBs by ferric ions, hydrogen peroxide and UV light," *Environ. Toxicol. Chem.*, vol. 13, no. 3, pp. 423–427, 1994.

[8] W. Gernjak, T. Krutzler, A. Glaser, et al., "Photo-Fenton treatment of water containing natural phenolic pollutants," *Chemosphere*, vol. 50, no. 1, pp. 71–78, 2003.

